# Supplementary material for: Associations between sleep habits, performance in reading and mathematics, and inattention and hyperactivity,
Source: PLoS One. 2026 May 20;21(5):e0347892. doi: 10.1371/journal.pone.0347892 (PMC13189340; doi:10.1371/journal.pone.0347892)

S1. Code and Output for Exploratory Factor Analyses

SleepEFA.R

slarsen3

2026-02-25

```
#EFA re-run 2025. This EFA examines the factor structure of the sleep items
```

```
Data2=read.csv("Cleaned_Sleep_Data_130223.csv", header=T)

library(psych)
library(tidyr)
library(dplyr)
```

```
##
## Attaching package: 'dplyr'
```

```
## The following objects are masked from 'package:stats':
##
##     filter, lag
```

```
## The following objects are masked from 'package:base':
##
##     intersect, setdiff, setequal, union
```

```
library(ggplot2)
```

```
##
## Attaching package: 'ggplot2'
```

```
## The following objects are masked from 'package:psych':
##
##     %+%, alpha
```

```
#Sleep item labels (grades indicated by G3, G5, G7, G9)

#G3_SleepDiff: Do the children have difficulty getting to sleep?
#G3_Wakeup: Do the children wake up more than twice per night?
#G3_Snore: Do the children snore?
#G3_Tired1: Do the children wake up in the morning and feel tired?
#G3_Tired2: Do the children experience daytime sleepiness?
#G3_Tired3: Do the children fall asleep during daytime activities
#(for example, in class, in conversation, watching TV, reading, riding in the car, etc)?
```

```
#EFA for sleep items
```

```
#Select out one twin at random - variable Rrandom is a random twin variable

Data2A <- Data2 [ which(Data2$Rrandom==1), ]

#Data2A <- Data2 [ which(Data1$Rrandom==2), ]

Data2A_G3 <- Data2A %>%
  select("G3_SleepDiff", "G3_Wakeup", "G3_Snore", "G3_Tired1", "G3_Tired2", "G3_Tired3" )


# Optional diagnostics
KMO(Data2A_G3)
```

```
## Kaiser-Meyer-Olkin factor adequacy
## Call: KMO(r = Data2A_G3)
## Overall MSA =  0.65
## MSA for each item =
## G3_SleepDiff    G3_Wakeup     G3_Snore    G3_Tired1    G3_Tired2    G3_Tired3
##         0.60         0.66         0.74         0.65         0.65         0.64
```

```
# Scree plot
fa.parallel(Data2A_G3, fa = "fa")
```

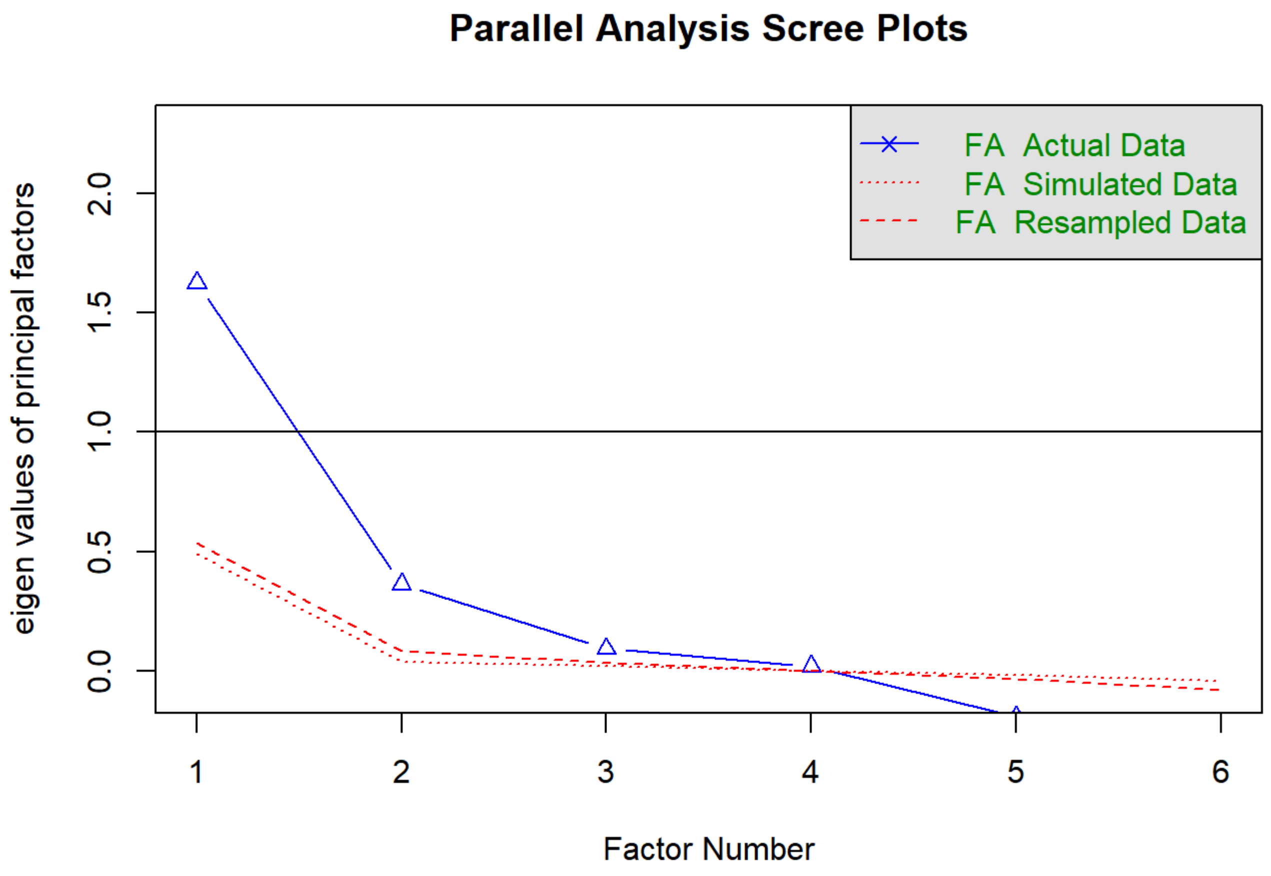


```
## Parallel analysis suggests that the number of factors =  4  and the number of components =
NA
```

```
# Run EFA
efa_result3 <- fa(Data2A_G3, nfactors = 2, rotate = "oblimin", fm = "ml")
```

```
## Loading required namespace: GPArotation
```

```
# Print and plot
print(efa_result3, digits = 2, sort = TRUE)
```

```
## Factor Analysis using method =  ml
## Call: fa(r = Data2A_G3, nfactors = 2, rotate = "oblimin", fm = "ml")
## Standardized loadings (pattern matrix) based upon correlation matrix
##              item   ML2   ML1    h2    u2 com
## G3_Tired2       5  0.86 -0.02 0.728 0.272 1.0
## G3_Tired3       6  0.54 -0.10 0.275 0.725 1.1
## G3_Tired1       4  0.50  0.30 0.427 0.573 1.6
## G3_Snore        3  0.25  0.02 0.063 0.937 1.0
## G3_SleepDiff    1 -0.01  1.00 0.995 0.005 1.0
## G3_Wakeup       2  0.15  0.34 0.167 0.833 1.4
##
##                        ML2  ML1
## SS loadings           1.40 1.25
## Proportion Var        0.23 0.21
## Cumulative Var        0.23 0.44
## Proportion Explained  0.53 0.47
## Cumulative Proportion 0.53 1.00
##
##  With factor correlations of
##      ML2  ML1
## ML2 1.00 0.29
## ML1 0.29 1.00
##
## Mean item complexity =  1.2
## Test of the hypothesis that 2 factors are sufficient.
##
## The degrees of freedom for the null model are  15  and the objective function was  1 with
Chi Square of  2803.01
## The degrees of freedom for the model are 4  and the objective function was  0.04
##
## The root mean square of the residuals (RMSR) is  0.04
## The df corrected root mean square of the residuals is  0.08
##
## The harmonic number of observations is  1439 with the empirical chi square  69.41  with pr
ob <  3e-14
## The total number of observations was  2818  with Likelihood Chi Square =  118.43  with pro
b <  1.2e-24
##
## Tucker Lewis Index of factoring reliability =  0.846
## RMSEA index =  0.101  and the 90 % confidence intervals are  0.086 0.117
## BIC =  86.66
## Fit based upon off diagonal values = 0.98
## Measures of factor score adequacy
##                                                    ML2  ML1
## Correlation of (regression) scores with factors   0.89 1.00
## Multiple R square of scores with factors          0.79 1.00
## Minimum correlation of possible factor scores     0.58 0.99
```

```
fa.diagram(efa_result3)
```

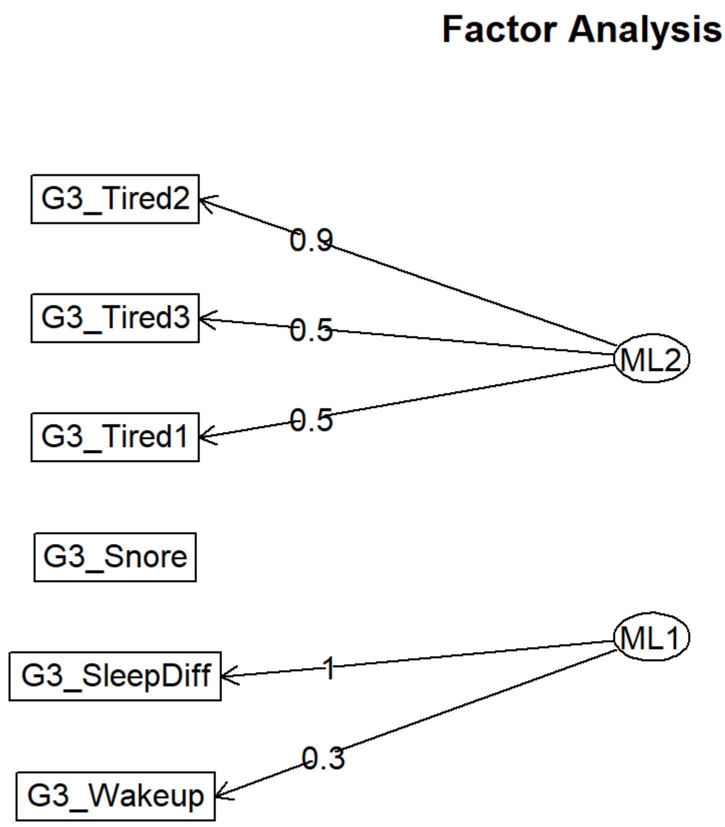


```
#Grade 5

Data2A_G5 <- Data2A %>%
  select("G5_SleepDiff", "G5_Wakeup", "G5_Snore", "G5_Tired1", "G5_Tired2", "G5_Tired3" )

# Optional diagnostics
KMO(Data2A_G5)
```

```
## Kaiser-Meyer-Olkin factor adequacy
## Call: KMO(r = Data2A_G5)
## Overall MSA =  0.66
## MSA for each item =
## G5_SleepDiff    G5_Wakeup     G5_Snore    G5_Tired1    G5_Tired2    G5_Tired3
##         0.66         0.70         0.82         0.68         0.65         0.60
```

```
# Scree plot
fa.parallel(Data2A_G5, fa = "fa")
```

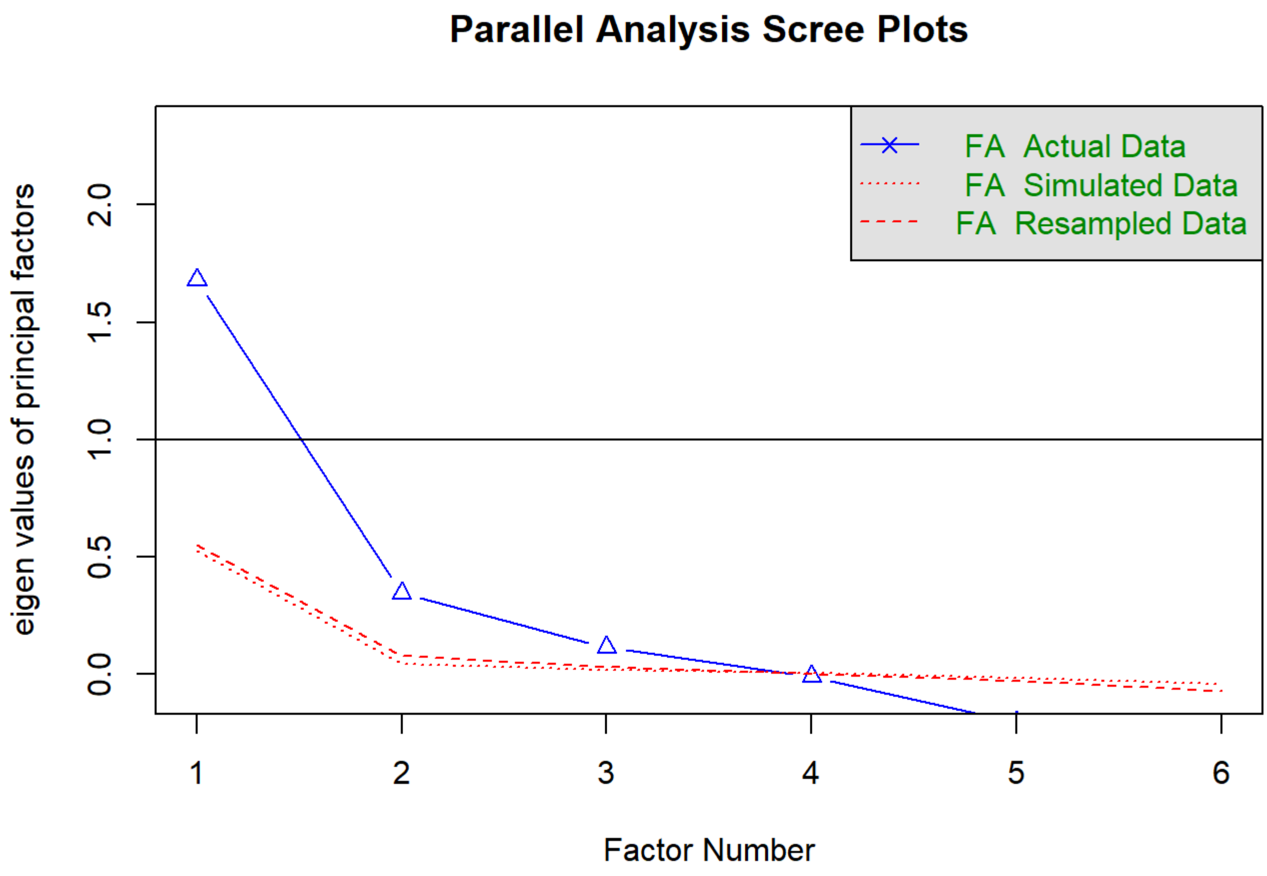


```
## Parallel analysis suggests that the number of factors =  3  and the number of components =
NA
```

```
# Run EFA
efa_result5 <- fa(Data2A_G5, nfactors = 2, rotate = "oblimin", fm = "ml")

# Print and plot
print(efa_result5, digits = 2, sort = TRUE)
```

```
## Factor Analysis using method =  ml
## Call: fa(r = Data2A_G5, nfactors = 2, rotate = "oblimin", fm = "ml")
## Standardized loadings (pattern matrix) based upon correlation matrix
##              item   ML1   ML2    h2    u2 com
## G5_Tired2       5  0.98 -0.01 0.947 0.053 1.0
## G5_Tired3       6  0.48 -0.06 0.219 0.781 1.0
## G5_Tired1       4  0.41  0.34 0.373 0.627 1.9
## G5_Snore        3  0.15  0.10 0.041 0.959 1.7
## G5_SleepDiff    1 -0.02  0.95 0.893 0.107 1.0
## G5_Wakeup       2  0.14  0.41 0.229 0.771 1.2
##
##                        ML1  ML2
## SS loadings           1.44 1.26
## Proportion Var        0.24 0.21
## Cumulative Var        0.24 0.45
## Proportion Explained  0.53 0.47
## Cumulative Proportion 0.53 1.00
##
##  With factor correlations of
##      ML1  ML2
## ML1 1.00 0.33
## ML2 0.33 1.00
##
## Mean item complexity =  1.3
## Test of the hypothesis that 2 factors are sufficient.
##
## The degrees of freedom for the null model are  15  and the objective function was  1.04 wi
th Chi Square of  2925.83
## The degrees of freedom for the model are 4  and the objective function was  0.02
##
## The root mean square of the residuals (RMSR) is  0.03
## The df corrected root mean square of the residuals is  0.06
##
## The harmonic number of observations is  1525 with the empirical chi square  42.68  with pr
ob <  1.2e-08
## The total number of observations was  2818  with Likelihood Chi Square =  67.19  with prob
<  8.9e-14
##
## Tucker Lewis Index of factoring reliability =  0.919
## RMSEA index =  0.075  and the 90 % confidence intervals are  0.06 0.091
## BIC =  35.41
## Fit based upon off diagonal values = 0.99
## Measures of factor score adequacy
##                                                    ML1  ML2
## Correlation of (regression) scores with factors   0.97 0.95
## Multiple R square of scores with factors          0.95 0.90
## Minimum correlation of possible factor scores     0.90 0.80
```

```
fa.diagram(efa_result5)
```

Factor Analysis

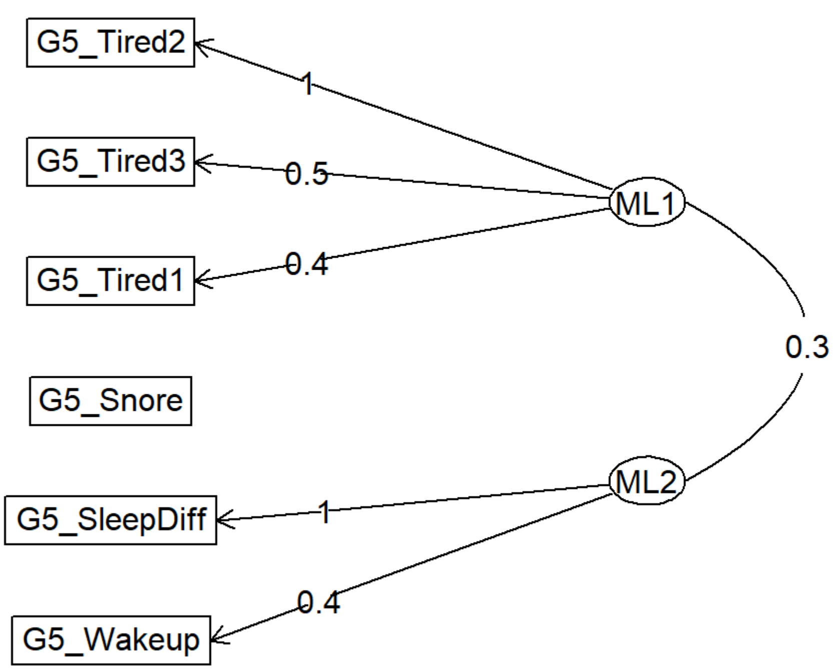


```
#Grade 7

Data2A_G7 <- Data2A %>%
  select("G7_SleepDiff", "G7_Wakeup", "G7_Snore", "G7_Tired1", "G7_Tired2", "G7_Tired3" )

# Optional diagnostics
KMO(Data2A_G7)
```

```
## Kaiser-Meyer-Olkin factor adequacy
## Call: KMO(r = Data2A_G7)
## Overall MSA =  0.67
## MSA for each item = 
## G7_SleepDiff    G7_Wakeup     G7_Snore    G7_Tired1    G7_Tired2    G7_Tired3 
##         0.67         0.72         0.85         0.67         0.65         0.63
```

```
# Scree plot
fa.parallel(Data2A_G7, fa = "fa")
```

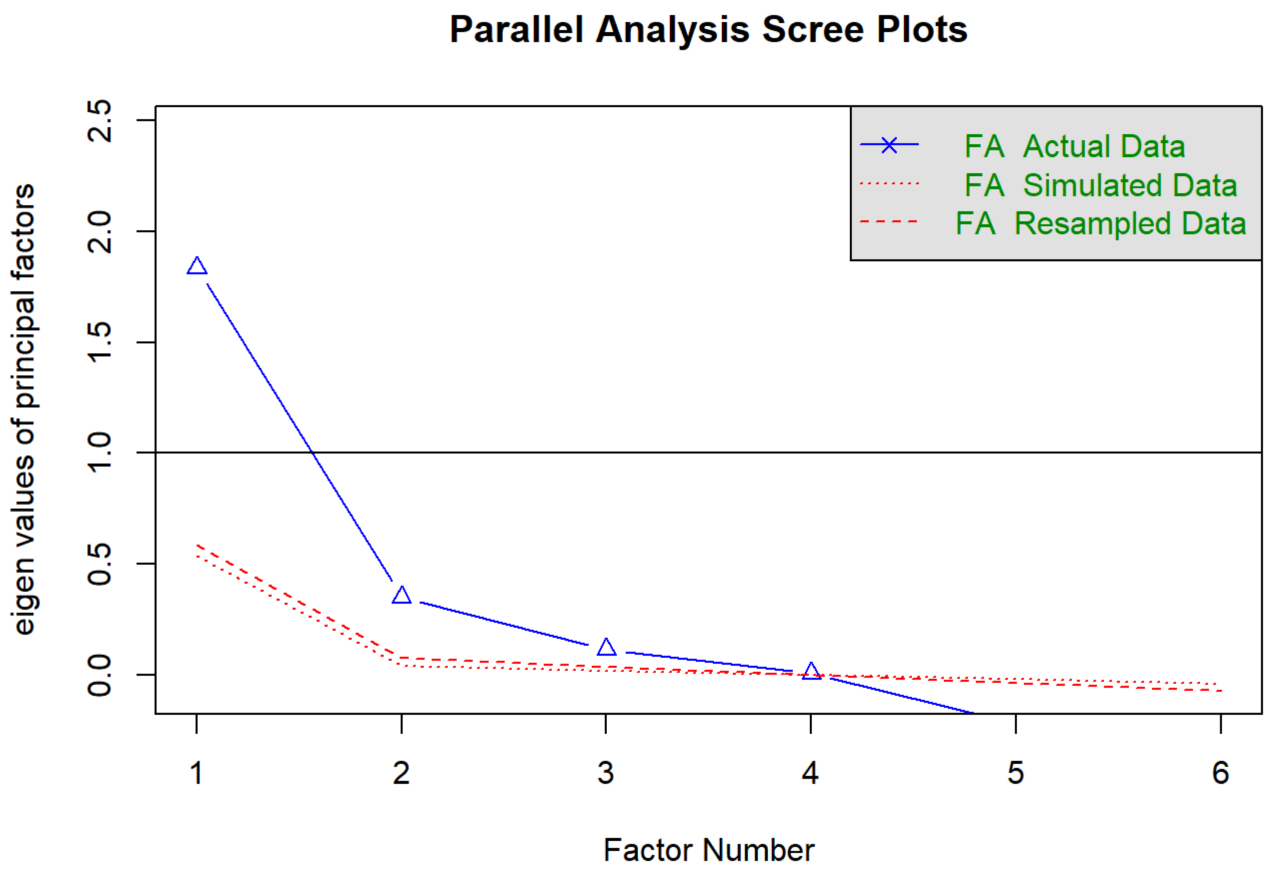


```
## Parallel analysis suggests that the number of factors =  3  and the number of components =
NA
```

```
# Run EFA
efa_result7 <- fa(Data2A_G7, nfactors = 2, rotate = "oblimin", fm = "ml")

# Print and plot
print(efa_result7, digits = 2, sort = TRUE)
```

```
## Factor Analysis using method =  ml
## Call: fa(r = Data2A_G7, nfactors = 2, rotate = "oblimin", fm = "ml")
## Standardized loadings (pattern matrix) based upon correlation matrix
##              item   ML1   ML2    h2    u2 com
## G7_Tired2       5  0.99 -0.02 0.969 0.031 1.0
## G7_Tired3       6  0.51 -0.02 0.253 0.747 1.0
## G7_Tired1       4  0.42  0.37 0.427 0.573 2.0
## G7_Snore        3  0.15  0.09 0.042 0.958 1.6
## G7_SleepDiff    1 -0.03  0.91 0.814 0.186 1.0
## G7_Wakeup       2  0.11  0.48 0.278 0.722 1.1
##
##                        ML1  ML2
## SS loadings           1.51 1.27
## Proportion Var        0.25 0.21
## Cumulative Var        0.25 0.46
## Proportion Explained  0.54 0.46
## Cumulative Proportion 0.54 1.00
##
##  With factor correlations of
##      ML1  ML2
## ML1 1.00 0.36
## ML2 0.36 1.00
##
## Mean item complexity =  1.3
## Test of the hypothesis that 2 factors are sufficient.
##
## The degrees of freedom for the null model are  15  and the objective function was  1.23 wi
th Chi Square of  3453.99
## The degrees of freedom for the model are 4  and the objective function was  0.05
##
## The root mean square of the residuals (RMSR) is  0.04
## The df corrected root mean square of the residuals is  0.08
##
## The harmonic number of observations is  1429 with the empirical chi square  70.75  with pr
ob <  1.6e-14
## The total number of observations was  2818  with Likelihood Chi Square =  130.66  with pro
b <  2.8e-27
##
## Tucker Lewis Index of factoring reliability =  0.862
## RMSEA index =  0.106  and the 90 % confidence intervals are  0.091 0.122
## BIC =  98.88
## Fit based upon off diagonal values = 0.98
## Measures of factor score adequacy
##                                                    ML1  ML2
## Correlation of (regression) scores with factors   0.98 0.92
## Multiple R square of scores with factors          0.97 0.84
## Minimum correlation of possible factor scores     0.94 0.68
```

```
fa.diagram(efa_result7)
```

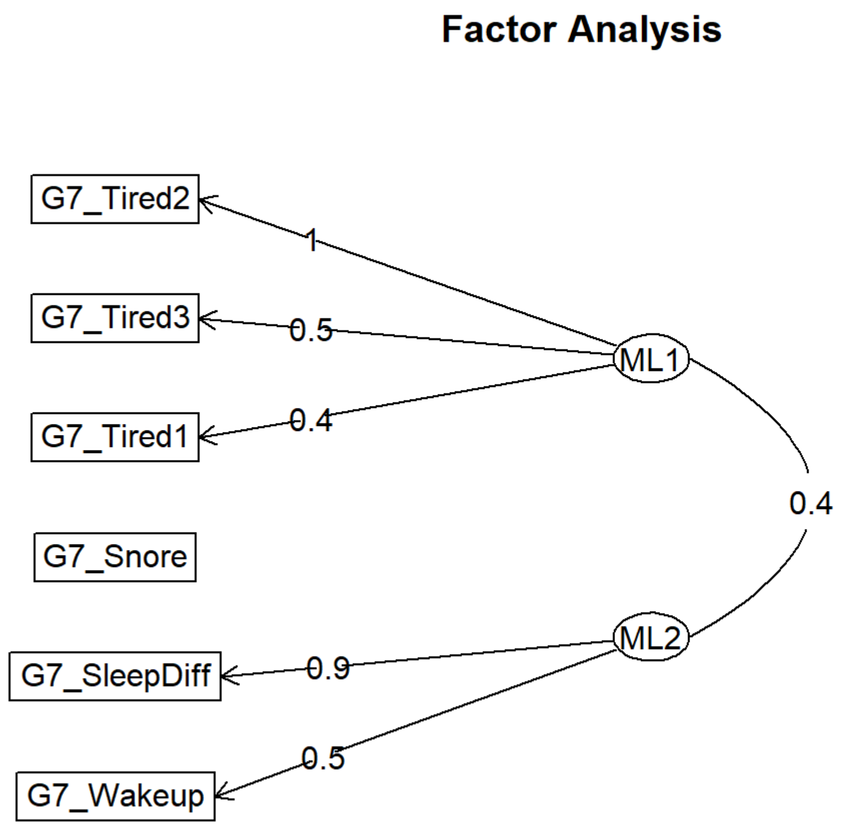


```
#Grade 9

Data2A_G9 <- Data2A %>%
  select("G9_SleepDiff", "G9_Wakeup", "G9_Snore", "G9_Tired1", "G9_Tired2", "G9_Tired3" )

# Optional diagnostics
KMO(Data2A_G9)
```

```
## Kaiser-Meyer-Olkin factor adequacy
## Call: KMO(r = Data2A_G9)
## Overall MSA =  0.67
## MSA for each item =
## G9_SleepDiff    G9_Wakeup     G9_Snore    G9_Tired1    G9_Tired2    G9_Tired3
##         0.69         0.71         0.78         0.67         0.63         0.62
```

```
# Scree plot
fa.parallel(Data2A_G9, fa = "fa")
```

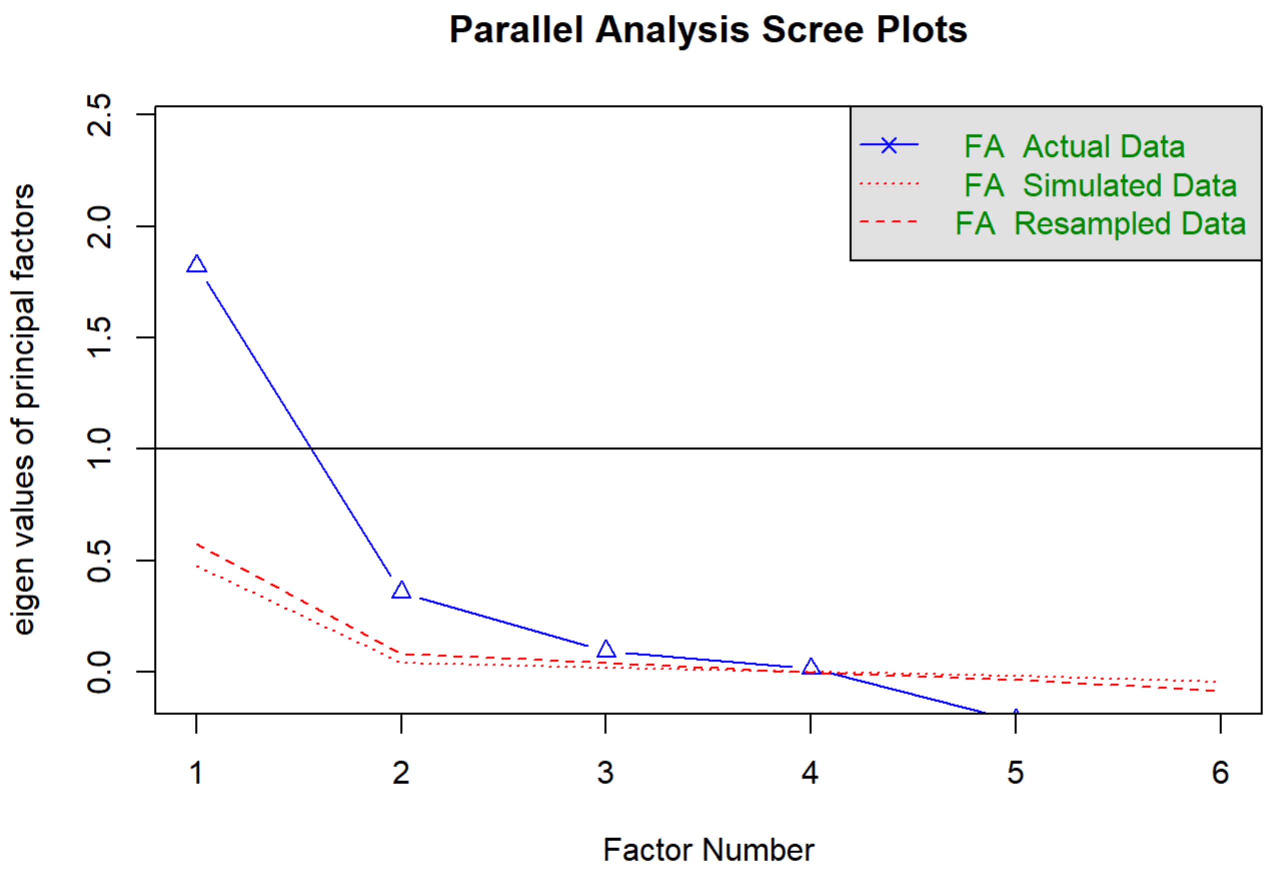


```
## Parallel analysis suggests that the number of factors =  4  and the number of components =
NA
```

```
# Run EFA
efa_result9 <- fa(Data2A_G9, nfactors = 2, rotate = "oblimin", fm = "ml")

# Print and plot
print(efa_result9, digits = 2, sort = TRUE)
```

```
## Factor Analysis using method =  ml
## Call: fa(r = Data2A_G9, nfactors = 2, rotate = "oblimin", fm = "ml")
## Standardized loadings (pattern matrix) based upon correlation matrix
##               item   ML1   ML2   h2    u2 com
## G9_Tired2       5  1.00 -0.02 1.00 0.005 1.0
## G9_Tired3       6  0.45  0.01 0.21 0.793 1.0
## G9_Tired1       4  0.42  0.40 0.47 0.525 2.0
## G9_SleepDiff    1 -0.02  0.76 0.56 0.444 1.0
## G9_Wakeup       2 -0.02  0.65 0.41 0.589 1.0
## G9_Snore        3  0.09  0.17 0.05 0.950 1.6
##
##                        ML1  ML2
## SS loadings           1.45 1.24
## Proportion Var        0.24 0.21
## Cumulative Var        0.24 0.45
## Proportion Explained  0.54 0.46
## Cumulative Proportion 0.54 1.00
##
##  With factor correlations of
##      ML1  ML2
## ML1 1.00 0.41
## ML2 0.41 1.00
##
## Mean item complexity =  1.3
## Test of the hypothesis that 2 factors are sufficient.
##
## The degrees of freedom for the null model are  15  and the objective function was  1.22 wi
th Chi Square of  3423.27
## The degrees of freedom for the model are 4  and the objective function was  0.04
##
## The root mean square of the residuals (RMSR) is  0.04
## The df corrected root mean square of the residuals is  0.07
##
## The harmonic number of observations is  1250 with the empirical chi square  48.26  with pr
ob <  8.3e-10
## The total number of observations was  2818  with Likelihood Chi Square =  120.01  with pro
b <  5.3e-25
##
## Tucker Lewis Index of factoring reliability =  0.872
## RMSEA index =  0.101  and the 90 % confidence intervals are  0.086 0.117
## BIC =  88.24
## Fit based upon off diagonal values = 0.99
## Measures of factor score adequacy
##                                                    ML1  ML2
## Correlation of (regression) scores with factors   1.00 0.85
## Multiple R square of scores with factors          0.99 0.72
## Minimum correlation of possible factor scores     0.99 0.43
```

```
fa.diagram(efa_result9)
```

Factor Analysis

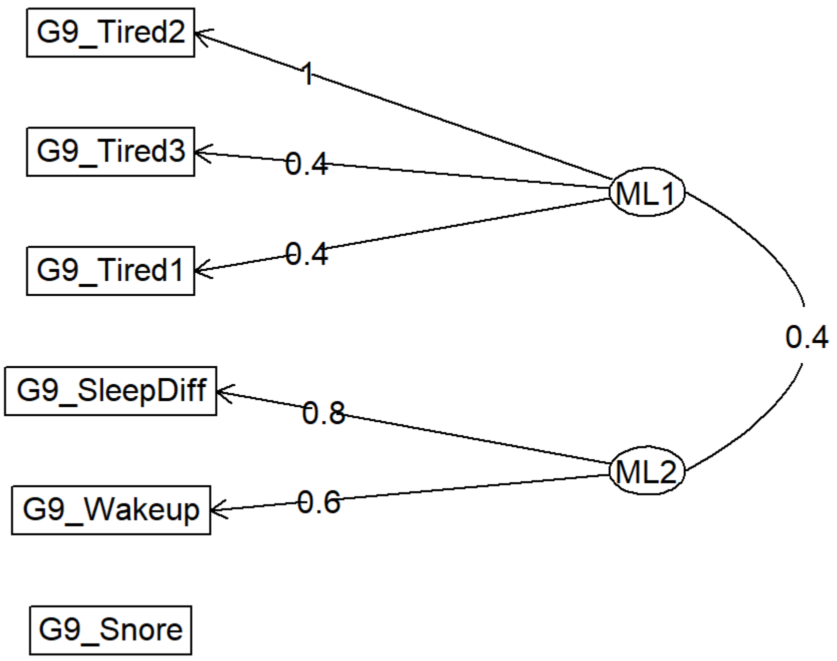

Supplement: S1 File — (PDF) [file pone.0347892.s001.pdf]
