# Supplementary material for: Associations between sleep habits, performance in reading and mathematics, and inattention and hyperactivity,
Source: PLoS One. 2026 May 20;21(5):e0347892. doi: 10.1371/journal.pone.0347892 (PMC13189340; doi:10.1371/journal.pone.0347892)
Supplement: S2 File — (DOCX) [file pone.0347892.s002.docx]

**S2.** Supplementary Tables

**Table. Mediation of Daytime Sleepiness Effect on Grade 3 Reading and Numeracy by Inattention and Hyperactivity.**

|  |  | **Total Effect** | | | **Direct Effect** | | |
| --- | --- | --- | --- | --- | --- | --- | --- |
|  |  | **Std Beta** | **Std SE** | **p** | **Std Beta** | **Std SE** | **p** |
| **Reading^a^** | Intercept | -0.084 | 0.052 | 0.108 | -0.008 | 0.048 | 0.866 |
|  | SES | 0.313 | 0.033 | <0.001 | 0.245 | 0.033 | <0.001 |
|  | Age | 0.107 | 0.035 | 0.003 | 0.063 | 0.032 | 0.048 |
|  | Sex | 0.087 | 0.058 | 0.134 | -0.048 | 0.056 | 0.386 |
|  | coTwin Sex | 0.080 | 0.058 | 0.171 | 0.064 | 0.055 | 0.244 |
|  | **Between-Pair Effects** | | | | | | |
|  | Regular Bedtime | -0.009 | 0.036 | 0.813 | -0.007 | 0.034 | 0.831 |
|  | Sleep on School Nights | 0.000 | 0.050 | 0.996 | -0.007 | 0.048 | 0.891 |
|  | Sleep on Weekends | 0.043 | 0.050 | 0.388 | 0.048 | 0.048 | 0.322 |
|  | Sleep Quality | 0.059 | 0.037 | 0.107 | 0.015 | 0.035 | 0.673 |
|  | Daytime Sleepiness | -0.018 | 0.039 | 0.644 | -0.056 | 0.036 | 0.117 |
|  | Snoring | -0.023 | 0.035 | 0.510 | -0.015 | 0.031 | 0.631 |
|  | Inattention |  |  |  | 0.387 | 0.049 | <0.001 |
|  | Hyperactivity |  |  |  | -0.059 | 0.050 | 0.244 |
|  | **Within-Pair Effects** | | | | | | |
|  | Regular Bedtime | 0.047 | 0.029 | 0.166 | 0.035 | 0.024 | 0.197 |
|  | Sleep on School Nights | 0.020 | 0.025 | 0.437 | 0.011 | 0.023 | 0.645 |
|  | Sleep on Weekends | 0.000 | 0.026 | 0.985 | 0.004 | 0.024 | 0.857 |
|  | Sleep Quality | -0.005 | 0.024 | 0.847 | -0.017 | 0.025 | 0.491 |
|  | Daytime Sleepiness | 0.039 | 0.021 | 0.068 | 0.014 | 0.021 | 0.485 |
|  | Snoring | -0.022 | 0.018 | 0.228 | -0.006 | 0.017 | 0.747 |
|  | Inattention |  |  |  | 0.123 | 0.025 | <0.001 |
|  | Hyperactivity |  |  |  | -0.012 | 0.028 | 0.679 |
| **Numeracy^b^** | Intercept | 0.110 | 0.056 | 0.050 | 0.189 | 0.051 | <0.001 |
|  | SES | 0.308 | 0.034 | <0.001 | 0.235 | 0.031 | <0.001 |
|  | Age | 0.097 | 0.036 | 0.008 | 0.049 | 0.032 | 0.125 |
|  | Sex | -0.226 | 0.058 | <0.001 | -0.389 | 0.055 | <0.001 |
|  | coTwin Sex | 0.009 | 0.057 | 0.870 | 0.013 | 0.054 | 0.804 |
|  | **Between-Pair Effects** | | | | | | |
|  | Regular Bedtime | -0.055 | 0.036 | 0.126 | -0.056 | 0.033 | 0.095 |
|  | Sleep on School Nights | -0.022 | 0.050 | 0.662 | -0.026 | 0.047 | 0.583 |
|  | Sleep on Weekends | 0.064 | 0.050 | 0.201 | 0.071 | 0.045 | 0.112 |
|  | Sleep Quality | 0.035 | 0.040 | 0.383 | -0.010 | 0.038 | 0.789 |
|  | Daytime Sleepiness | 0.009 | 0.040 | 0.812 | -0.033 | 0.035 | 0.350 |
|  | Snoring | -0.024 | 0.038 | 0.539 | -0.018 | 0.035 | 0.609 |
|  | Inattention |  |  |  | 0.487 | 0.046 | <0.001 |
|  | Hyperactivity |  |  |  | -0.151 | 0.049 | 0.003 |
|  | **Within-Pair Effects** | | | | | | |
|  | Regular Bedtime | 0.008 | 0.032 | 0.816 | -0.012 | 0.022 | 0.596 |
|  | Sleep on School Nights | -0.002 | 0.027 | 0.943 | -0.014 | 0.023 | 0.542 |
|  | Sleep on Weekends | 0.004 | 0.025 | 0.882 | 0.007 | 0.021 | 0.738 |
|  | Sleep Quality | 0.032 | 0.022 | 0.137 | 0.015 | 0.021 | 0.474 |
|  | Daytime Sleepiness | 0.038 | 0.018 | 0.036 | 0.005 | 0.018 | 0.796 |
|  | Snoring | -0.029 | 0.018 | 0.107 | -0.008 | 0.018 | 0.653 |
|  | Inattention |  |  |  | 0.154 | 0.024 | <0.001 |
|  | Hyperactivity |  |  |  | 0.007 | 0.023 | 0.746 |

^a^ *N* = 1246; ^b^ *N* = 1244

**Table. Mediation of Sleep Quality Effect on Grade 9 Reading by Inattention and Hyperactivity.**

|  |  | **Total Effect** | | | **Direct Effect** | | |
| --- | --- | --- | --- | --- | --- | --- | --- |
|  |  | **Std Beta** | **Std SE** | **p** | **Std Beta** | **Std SE** | **p** |
| **Reading^a^** | Intercept | -0.005 | 0.058 | 0.928 | 0.082 | 0.054 | 0.130 |
|  | SES | 0.326 | 0.035 | <0.001 | 0.272 | 0.032 | <0.001 |
|  | Age | 0.024 | 0.034 | 0.483 | 0.006 | 0.032 | 0.842 |
|  | Sex | 0.061 | 0.064 | 0.340 | -0.104 | 0.063 | 0.096 |
|  | coTwin Sex | -0.049 | 0.064 | 0.443 | -0.057 | 0.063 | 0.363 |
|  | **Between-Pair Effects** | | | | | | |
|  | Regular Bedtime | -0.002 | 0.038 | 0.951 | -0.007 | 0.033 | 0.840 |
|  | Sleep on School Nights | -0.037 | 0.043 | 0.388 | -0.030 | 0.039 | 0.437 |
|  | Sleep on Weekends | 0.051 | 0.040 | 0.204 | 0.046 | 0.036 | 0.201 |
|  | Sleep Quality | 0.058 | 0.044 | 0.190 | 0.037 | 0.038 | 0.332 |
|  | Daytime Sleepiness | -0.088 | 0.042 | 0.040 | -0.139 | 0.040 | 0.001 |
|  | Snoring | 0.126 | 0.040 | 0.002 | 0.105 | 0.034 | 0.003 |
|  | Inattention |  |  |  | 0.408 | 0.054 | <0.001 |
|  | Hyperactivity |  |  |  | -0.088 | 0.051 | 0.089 |
|  | **Within-Pair Effects** | | | | | | |
|  | Regular Bedtime | 0.020 | 0.020 | 0.330 | 0.008 | 0.020 | 0.699 |
|  | Sleep on School Nights | -0.066 | 0.022 | 0.005 | -0.062 | 0.021 | 0.005 |
|  | Sleep on Weekends | -0.016 | 0.019 | 0.391 | -0.012 | 0.019 | 0.526 |
|  | Sleep Quality | 0.063 | 0.022 | 0.004 | 0.045 | 0.022 | 0.047 |
|  | Daytime Sleepiness | -0.003 | 0.024 | 0.891 | -0.016 | 0.024 | 0.491 |
|  | Snoring | 0.035 | 0.019 | 0.075 | 0.034 | 0.019 | 0.072 |
|  | Inattention |  |  |  | 0.127 | 0.029 | <0.001 |
|  | Hyperactivity |  |  |  | -0.035 | 0.033 | 0.301 |
| **Numeracy^b^** | Intercept | 0.117 | 0.058 | 0.045 | 0.219 | 0.052 | <0.001 |
|  | SES | 0.370 | 0.035 | <0.001 | 0.313 | 0.031 | <0.001 |
|  | Age | 0.021 | 0.038 | 0.584 | -0.002 | 0.032 | 0.939 |
|  | Sex | -0.263 | 0.063 | <0.001 | -0.492 | 0.057 | <0.001 |
|  | coTwin Sex | 0.038 | 0.063 | 0.543 | 0.068 | 0.057 | 0.236 |
|  | **Between-Pair Effects** | | | | | | |
|  | Regular Bedtime | -0.004 | 0.034 | 0.906 | -0.003 | 0.031 | 0.911 |
|  | Sleep on School Nights | -0.002 | 0.039 | 0.952 | 0.004 | 0.034 | 0.898 |
|  | Sleep on Weekends | -0.032 | 0.038 | 0.398 | -0.036 | 0.034 | 0.300 |
|  | Sleep Quality | 0.105 | 0.039 | 0.008 | 0.086 | 0.034 | 0.012 |
|  | Daytime Sleepiness | -0.033 | 0.042 | 0.425 | -0.096 | 0.038 | 0.012 |
|  | Snoring | 0.113 | 0.036 | 0.002 | 0.091 | 0.030 | 0.003 |
|  | Inattention |  |  |  | 0.529 | 0.051 | <0.001 |
|  | Hyperactivity |  |  |  | -0.181 | 0.051 | 0.001 |
|  | **Within-Pair Effects** | | | | | | |
|  | Regular Bedtime | 0.026 | 0.021 | 0.238 | 0.005 | 0.020 | 0.792 |
|  | Sleep on School Nights | -0.024 | 0.022 | 0.275 | -0.015 | 0.019 | 0.427 |
|  | Sleep on Weekends | -0.034 | 0.021 | 0.115 | -0.027 | 0.022 | 0.231 |
|  | Sleep Quality | 0.045 | 0.019 | 0.022 | 0.010 | 0.018 | 0.566 |
|  | Daytime Sleepiness | 0.037 | 0.022 | 0.094 | 0.016 | 0.021 | 0.448 |
|  | Snoring | 0.037 | 0.019 | 0.048 | 0.034 | 0.019 | 0.074 |
|  | Inattention |  |  |  | 0.189 | 0.025 | <0.001 |
|  | Hyperactivity |  |  |  | -0.021 | 0.024 | 0.402 |

^a^ *N* = 1103; ^b^ *N* = 1096
