# Supplementary material for: Associations between sleep habits, performance in reading and mathematics, and inattention and hyperactivity,
Source: PLoS One. 2026 May 20;21(5):e0347892. doi: 10.1371/journal.pone.0347892 (PMC13189340; doi:10.1371/journal.pone.0347892)
Supplement: Attachment — Submitted filename: Reviewer Report.docx [file pone.0347892.s004.docx]

**Reviewer Report**

**Manuscript Title:** *Associations between Sleep Habits, Performance in Reading and Mathematics, and Inattention and Hyperactivity*

**Appreciation of the Work and Principal Investigators**

I commend the authors, led by a highly experienced and internationally recognized team of investigators, for conducting and reporting on a large-scale, longitudinal twin study. The sample size, design, and rigorous statistical approach make this a rare and valuable contribution to the literature. The integration of standardized academic achievement measures (NAPLAN), validated parental reports of sleep and ADHD symptoms, and the twin design substantially enhance the study’s internal validity.

The manuscript reflects thorough planning, clear reporting, and transparent disclosure of conflicts of interest and funding. It aligns well with the open-science values of *PLOS ONE*. The commitment to methodological rigor such as between- and within-pair modelling, multiple testing correction, and careful attention to potential confounders—demonstrates a high level of scientific responsibility.

This study enriches the field by examining three interconnected domains—sleep, academic performance, and ADHD-related behaviors in the same dataset, rather than isolating them. Such integrative work is essential for moving beyond siloed findings.

**Minor Issues**

1. **Abstract presentation**: The abstract would benefit from more explicit effect size reporting in percentage terms, helping readers quickly understand the limited practical significance of the findings.
2. **Terminology consistency**: Terms such as “sleep habits” and “sleep patterns” are used interchangeably. Standardizing terminology would aid clarity.
3. **Tables**: The main text tables are extremely detailed. For readability, consider moving some of the more complex regression tables to supplementary materials while retaining the core findings in the manuscript.
4. **Unexpected results**: Certain results (e.g., better sleep quality predicting lower reading in Grade 7, snoring predicting better attention in Grade 3) are acknowledged but only briefly. A clearer explanation (or acknowledgment as possible artifacts) should be provided.
5. **References**: Some cited meta-analyses are over a decade old. Including more recent systematic reviews or meta-analyses on sleep and learning outcomes would strengthen the background.

**Major Issues**

1. **Interpretation of Effect Sizes**
   - Although the study correctly reports that sleep accounts for ~1% of variance in academic outcomes, more emphasis is needed on the limited *practical significance*. For educators and clinicians, these effects may be negligible. Conversely, the 3–7% explained variance in ADHD symptoms is potentially more meaningful and should be highlighted with greater nuance.
2. **Retrospective Reporting Bias**
   - Up to half of the parent sleep reports were retrospective, raising concerns about recall bias. The manuscript mentions this but does not discuss in depth how it may have influenced within or between pair differences.
3. **Counterintuitive and Inconsistent Findings**
   - Some associations were opposite to expectations. While chance findings due to multiple comparisons are plausible, the discussion should explicitly contextualize these outcomes, rather than presenting them as isolated anomalies.
4. **Sex Effects**
   - Although sex was tested and not retained as a moderator, prior research suggests sex differences in both sleep and ADHD expression. A sensitivity analysis or clearer justification for excluding sex moderation would strengthen confidence in the generalizability of the results.
5. **Mediation Analyses**
   - The mediation of sleep → inattention → academic outcomes was observed only in Grades 3 and 9. The discussion should address why mediation was inconsistent across grades, considering developmental, sample attrition, or measurement factors.

**Recommendations**

- **Strengthen interpretation**: Explicitly frame the findings as small, statistically significant but practically limited effects on academic outcomes, while highlighting somewhat stronger implications for ADHD-related behaviors.
- **Address retrospective data**: Provide a fuller discussion of recall bias and its possible influence on effect estimates.
- **Clarify unexpected results**: Offer stronger explanations or cautionary notes regarding counterintuitive findings (e.g., possible chance effects, measurement error).
- **Revisit sex differences**: Either test moderation more thoroughly or justify why sex effects can be discounted in this dataset.
- **Streamline presentation**: Move some of the very detailed regression outputs to supplementary material, keeping the manuscript accessible to the broader *PLOS ONE* readership.
- **Update literature**: Cite newer reviews or meta-analyses on sleep and learning/ADHD where available.

**Overall Recommendation**

**Major Revision**

The manuscript is methodologically sound and represents an important contribution, especially because of its large-scale twin design and simultaneous focus on sleep, ADHD, and academic outcomes. However, revisions are needed to strengthen interpretation, better address biases, and improve clarity. If these are addressed, the paper will provide a valuable and trustworthy addition to the field.

**Reviewer:**

Dr Fathi Ali Araye	Date : 25-Sept-2025

Psychiatrist
