# Supplementary material for: Associations between sleep habits, performance in reading and mathematics, and inattention and hyperactivity,
Source: PLoS One. 2026 May 20;21(5):e0347892. doi: 10.1371/journal.pone.0347892 (PMC13189340; doi:10.1371/journal.pone.0347892)
Supplement: Attachment — Submitted filename: Response to Reviewers_13Mar26.docx [file pone.0347892.s006.docx]

**PONE-D-25-42491**

**Associations between Sleep Habits, Performance in Reading and Mathematics, and Inattention and Hyperactivity**

**PLOS One**

Reviewer's Responses to Questions and Comments

Our responses to each of the queries raised by reviewers are noted in blue font below each item. Thank you for the insightful suggestions and comments.

Reviewer #1:

The manuscript is methodologically sound and represents an important contribution, especially because of its large-scale twin design and simultaneous focus on sleep, ADHD, and academic outcomes. However, revisions are necessary to strengthen the interpretation, better address biases and enhance clarity. If these are addressed, the paper will provide a valuable and trustworthy addition to the field.

**Manuscript Title:** *Associations between Sleep Habits, Performance in Reading and Mathematics, and Inattention and Hyperactivity*

**Appreciation of the Work and Principal Investigators**

I commend the authors, led by a highly experienced and internationally recognized team of investigators, for conducting and reporting on a large-scale, longitudinal twin study. The sample size, design, and rigorous statistical approach make this a rare and valuable contribution to the literature. The integration of standardized academic achievement measures (NAPLAN), validated parental reports of sleep and ADHD symptoms, and the twin design substantially enhance the study’s internal validity.

The manuscript reflects thorough planning, clear reporting, and transparent disclosure of conflicts of interest and funding. It aligns well with the open-science values of *PLOS ONE*. The commitment to methodological rigor such as between- and within-pair modelling, multiple testing correction, and careful attention to potential confounders—demonstrates a high level of scientific responsibility.

This study enriches the field by examining three interconnected domains—sleep, academic performance, and ADHD-related behaviors in the same dataset, rather than isolating them. Such integrative work is essential for moving beyond siloed findings.

Thank you for this feedback, it is very much appreciated.

**Minor Issues**

1. **Abstract presentation**: The abstract would benefit from more explicit effect size reporting in percentage terms, helping readers quickly understand the limited practical significance of the findings.

We have retained the percentage of variance explained in the abstract. The calculation of these percentages is explained on page 12. In the methods section. We have elaborated on the interpretation of these percentages and their practical implications in the discussion (also see Point 1 under Major Issues below for additional clarification).

2. **Terminology consistency**: Terms such as “sleep habits” and “sleep patterns” are used interchangeably. Standardizing terminology would aid clarity.

Thank you for this suggestion. We have clarified the terminology to align with existing sleep research, and have outlined the terms used at the end of the first paragraph of the introduction (p.3). We acknowledge that *sleep habits* refers to behaviours at sleep time (e.g. bedtime regularity) and *sleep patterns* refers to specific subcomponents of sleep physiology. We have also clarified these two terms in the Methods section where we describe the survey items relating to sleep (pp. 8-9).

3. **Tables**: The main text tables are extremely detailed. For readability, consider moving some of the more complex regression tables to supplementary materials while retaining the core findings in the manuscript.

Thank you for this suggestion and we acknowledge that the results are quite complex given the four age-grouped samples in the study. To aid in readability, we have moved Tables 7 and 8 pertaining to the post-hoc analyses, to the Supplementary Materials. We have also focused the results on only the standardised Beta and SE for each outcome. We hope this alteration helps retain the focus on the core findings of the main results while not omitting important details.

4. **Unexpected results**: Certain results (e.g., better sleep quality predicting lower reading in Grade 7, snoring predicting better attention in Grade 3) are acknowledged but only briefly. A clearer explanation (or acknowledgment as possible artifacts) should be provided.

This issue is closely related to the major concerns detailed below regarding counterintuitive and inconsistent findings.

However, that response does not address the unusual zero-order correlation finding in Grade 7. We have now included the below in the discussion to acknowledge that more explicitly:

“*This is an anomalous result when compared to the other grades and no association was evident in the between and within models. The only significant finding in the between and within-models was in Grade 9 when within-pair sleep quality was associated with better reading in the expected direction. Nonetheless, this effect disappeared when inattention was included. Thus we suspect that the zero order negative correlation between sleep quality and Grade 7 reading was biased by unmeasured confounders or influential cases that were accounted for in the full regression models but not in the correlations.*”(p.27-28)

5. **References**: Some cited meta-analyses are over a decade old. Including more recent systematic reviews or meta-analyses on sleep and learning outcomes would strengthen the background.

Thank you for this suggestion. We have cited the systematic review and meta-analysis by Mushaffen et al. (2021) along with an older review (Dewald et al., 2010). The former is the most relevant to the current study, given that it focuses on the associations between sleep and academic performance in adolescents, though the older paper gives additional context. We have now also included reference to Marten et al. (2023) and Xian et al. (2025), both systematic review and meta-analyses examining associations between sleep problems and ADHD in children and adolescents. We have also cited Sun et al. (2019) whose systematic review examined whether differences in sleep duration on weekdays and weekends were associated with academic and health outcomes in adolescents.

**Major Issues**

1. **Interpretation of Effect Sizes**
   - Although the study correctly reports that sleep accounts for ~1% of variance in academic outcomes, more emphasis is needed on the limited *practical significance*. For educators and clinicians, these effects may be negligible. Conversely, the 3–7% explained variance in ADHD symptoms is potentially more meaningful and should be highlighted with greater nuance.

Thank you for this suggestion. We have added to the discussion to highlight the practical implications of the variance in ADHD outcomes explained by the models (p.28). To put these effect sizes in context, we include a reference to a systematic review by Claussen et al. (2022), and we include a reference to a recent paper by Funder & Ozer (2019) to interpret these effects.

2. **Retrospective Reporting Bias**
   - Up to half of the parent sleep reports were retrospective, raising concerns about recall bias. The manuscript mentions this but does not discuss in depth how it may have influenced within or between pair differences.

This is a challenging aspect of these data. The actual proportion of retrospective sleep data for each grade was .40, .33, .32, and .28 for Grades 3, 5, 7, and 9 respectively. If parents systematically reported their twins to either be more or less similar when reported retrospectively compared to concurrently then this could impact our results for the academic outcome analyses. This is not a concern for the analyses that included ADHD as those data were only collected concurrently. To test if the retrospective reporting impacted our findings we selected one twin from each pair (to avoid the duplicate entry for each family as within pair measures are just a mirror of their twin) and conducted z-score difference tests on the between and within sleep measures for each grade.

No within-pair sleep variables were significantly different ( p < .05) when comparing retrospective reporting to concurrent reporting. However, there was a tendency for the family average to be retrospectively reported as sleeping slightly better than the concurrent reports, particularly in the younger Grades. Thus we re-ran the main analyses for reading and numeracy including a covariate for retrospectively collected parental sleep report. These results are now reported in the paper.

3. **Counterintuitive and Inconsistent Findings**
   - Some associations were opposite to expectations. While chance findings due to multiple comparisons are plausible, the discussion should explicitly contextualize these outcomes, rather than presenting them as isolated anomalies.

Following your suggestion to include a formal test of sex moderation, we adopted a recently developed approach, clubSandwich, (Pustejovsky, 2026) and the lme4 package in R to obtain robust sandwich standard errors for the mixed models. This method formally accounts for potential influential cases and the clustered twin structure, thereby removing the need for subjective decisions about data inclusion or exclusion. As a result, the robustness and reproducibility of our findings are substantially improved. We cite Bates et al. (2015) for the lme4 package – we had omitted to include this citation previously.

Notably, some of the previously observed unusual or counterintuitive results (such as better sleep quality predicting lower reading scores or snoring predicting better attention) did not remain statistically significant under this more rigorous approach. We interpret this as evidence that these findings were likely artifacts driven by influential data points rather than true effects, and we have accordingly adjusted our interpretation to focus on results that are more robust.

The remaining surprising findings—the association between between-pair hyperactivity and poorer numeracy—were already investigated and discussed in detail in the manuscript.

4. **Sex Effects**
   - Although sex was tested and not retained as a moderator, prior research suggests sex differences in both sleep and ADHD expression. A sensitivity analysis or clearer justification for excluding sex moderation would strengthen confidence in the generalizability of the results.

We conducted sex moderation analyses for all sleep variables for each outcome. None of the sex moderation effects reached significance, supporting the focus on the more parsimonious models without sex moderation. We have removed reference to our simpler method for assessing sex moderation, updating the manuscript to detail our formal test. (p.17)

5. **Mediation Analyses**
   - The mediation of sleep → inattention → academic outcomes was observed only in Grades 3 and 9. The discussion should address why mediation was inconsistent across grades, considering developmental, sample attrition, or measurement factors.

This is an interesting point. We would disagree that the mediation was inconsistent across grades. Quite the opposite. The mediation is similar across the grades. Formal mediation and tests of indirect effects were conducted only in Grades 3 and 9 because these were the only grades in which the within-pair association between a sleep predictor and the outcome was significant for both the academic outcomes and the ADHD outcome. In Grades 5 and 7, the total effects of specific within-pair sleep predictors on the academic outcomes were not significant, and standard mediation logic dictates that an indirect effect cannot be meaningfully tested in the absence of a total effect.

Additionally, the sleep predictors differed across the two grades with observed effects: daytime sleepiness in Grade 3 and sleep quality in Grade 9. Thus, the mediation effects are not inconsistent per se but may reflect the developmental specificity of which sleep factors relate to attention and academic performance at these grades.

The question then becomes, why is daytime sleepiness relevant to attention particularly in Grade 3 and why is sleep quality relevant to attention particularly in Grade 9. For the standardised estimates of within-pair daytime sleepiness and sleep quality across the grades,

- daytime sleepiness is passing nominal significance (.05) in all other grades but only passes multiple testing criterion in Grade 3, and,
- sleep quality passes nominal significance in Grade 3 and multiple testing criterion only in Grade 9.

This implies that the strength of these effects may differ with development, but are likely present to some degree throughout childhood and adolescence.

**Recommendations**

- **Strengthen interpretation**: Explicitly frame the findings as small, statistically significant but practically limited effects on academic outcomes, while highlighting somewhat stronger implications for ADHD-related behaviors.
- **Address retrospective data**: Provide a fuller discussion of recall bias and its possible influence on effect estimates.
- **Clarify unexpected results**: Offer stronger explanations or cautionary notes regarding counterintuitive findings (e.g., possible chance effects, measurement error).
- **Revisit sex differences**: Either test moderation more thoroughly or justify why sex effects can be discounted in this dataset.
- **Streamline presentation**: Move some of the very detailed regression outputs to supplementary material, keeping the manuscript accessible to the broader *PLOS ONE* readership.
- **Update literature**: Cite newer reviews or meta-analyses on sleep and learning/ADHD where available. Done

Thank you for all these recommendations. We have completed each of these in our revision of the paper.

**Overall Recommendation**

**Major Revision**

The manuscript is methodologically sound and represents an important contribution, especially because of its large-scale twin design and simultaneous focus on sleep, ADHD, and academic outcomes. However, revisions are needed to strengthen interpretation, better address biases, and improve clarity. If these are addressed, the paper will provide a valuable and trustworthy addition to the field.

**Reviewer:**

Dr Fathi Ali Araye	Date : 25-Sept-2025

Psychiatrist

**Reviewer #2:**

This manuscript examines associations between several aspects of sleep habits and academic performance (reading and mathematics), as well as inattention and hyperactivity, in a large sample of Australian twins. The use of nationwide standardized academic assessments and a twin-based between–within modeling approach are notable strengths. Overall, the main conclusions—namely that associations between sleep habits and academic outcomes are generally small, while relations with inattention/hyperactivity are more consistent—are supported by the data.

Thank you for this generous feedback.

Comments and suggestions for improvement
**1. Sleep measurement and retrospective reporting**
A proportion of the sleep data was collected retrospectively. While this is understandable in a longitudinal design, it would be helpful if the authors could briefly clarify how retrospective sleep reporting was implemented and acknowledge more explicitly how this may have influenced the results.

If feasible, a short sensitivity analysis or descriptive comparison between retrospective and concurrent reports would further strengthen the paper.

Thank you. This same issue was raised by Reviewer 1, which we addressed as follows:

This is a challenging aspect of these data. The actual proportion of retrospective sleep data for each grade was .40, .33, .32, and .28 for Grades 3, 5, 7, and 9 respectively. If parents systematically reported their twins to either be more or less similar when reported retrospectively compared to concurrently then this could impact our results for the academic outcome analyses. This is not a concern for the analyses that included ADHD as those data were only collected concurrently. To test if the retrospective reporting impacted our findings we selected one twin from each pair (to avoid the duplicate entry for each family as within pair measures are just a mirror of their twin) and conducted z-score difference tests on the between and withing sleep measures for each grade.

No within pair sleep variables were significantly different ( p < .05) when comparing retrospective reporting to concurrent reporting. However, there was a tendency for the family average to be retrospectively reported as sleeping slightly better than the concurrent reports, particularly in the younger Grades. Thus we re-ran the main analyses for reading and numeracy including a covariate for retrospectively collected parental sleep report. These results are now reported in the paper. (see Tables 3 and 4, pp. 18-21)

**2. Parent-reported measures**
Both sleep habits and inattention/hyperactivity were reported by parents, which may contribute to somewhat stronger associations between these domains compared with academic outcomes. This point is already noted in the limitations section, but a slightly expanded discussion would be helpful for readers.

Thank you for this suggestion. We have added some detail to the Limitations to expand on the possible biasing effects of common method variance. We also include a citation to Lance (2024) *A primer on method effects on observed correlations with examples*. We hope these additions further clarify this limitation.

**3. Sleep habit composites**
The selection of specific items from the Children’s Sleep Habits Questionnaire and the creation of composite measures for sleep quality and daytime sleepiness are reasonable. Providing a small amount of additional detail on the factor-analytic justification for these composites (for example, in supplementary material) would improve transparency and reproducibility.
Thank you for this suggestion. As reported in the paper, we had undertaken Exploratory Factor Analyses (EFA) on the items that were combined into the composite measures termed *sleep quality* and *daytime sleepiness.* We provide the analytic code and output for these analyses in the Supplementary Materials and have rewritten the explanation of our procedure for the EFA and the process of combining the variables into composites in the Methods section (p.8-9). In short, the combination of the variables into the composites was guided both by the domains reported in the Child Sleep Habits Questionnaire (CSHQ) from where we drew the items, and the results of the EFA.

**4. Within-pair effects**
The between–within modeling approach is a major strength of the study. For some sleep variables that show very high similarity within twin pairs (e.g., bedtime regularity), it may be useful to note that limited within-pair variability could reduce the precision of within-pair estimates, and therefore, such effects should be interpreted cautiously.

This is an excellent point. We have now discussed this in additional depth:

“*These high correlations impact the within-pair variance and the predictive power of this variable, but it does not necessarily reduce the between-pair variability. However, many families in out sample did endorse a high level of bedtime regularity. We a priori decided on the variables to include in our model, in future, it might be well worth also deciding on a minimum amount of variation in a predictor to include it in the models. It may be that bedtime regularity was a non-significant predictor in out analyses not because it is intrinsically unimportant, but because of ceiling effects.”* (p.29-30)

**5. Unexpected or inconsistent findings**
A small number of associations were in an unexpected direction or varied across grades. The authors appropriately note the overall inconsistency of effects; a brief additional comment suggesting possible explanations (e.g., measurement issues or statistical suppression) would further aid interpretation.

Following Reviewer 1’s suggestion to include a formal test of sex moderation, we adopted a recently developed approach, clubSandwich (Pustejovsky, 2026) to obtain robust sandwich standard errors for the mixed models. This approach formally accounts for potential influential cases and clustered data, removing the need for subjective decisions about data inclusion or exclusion. Importantly, this method improves the robustness and reproducibility of our results. Because of this, some of these unusual findings were no longer evident.

**6. Data availability**
The authors clearly state that reading and mathematics data are owned by Australian state and territory governments and cannot be shared publicly. It may be helpful to briefly indicate whether and how qualified researchers can request access to these data through the relevant authorities.
Thank you, we have added the relevant information about data availability as part of the submission process.

Summary
In summary, this is a well-conducted study with a strong design and a large, informative dataset. Addressing the points above would further clarify the methodology and interpretation, but the overall findings are sound and consistent with much of the existing literature.

Thank you for this positive feedback. It is much appreciated.
